# Oral parafunctional behaviors, TMD pain, and headaches among patients underwent orthodontic therapy—an observational study

**DOI:** 10.3389/fneur.2025.1548138

**Published:** 2025-04-07

**Authors:** Małgorzata Gałczyńska-Rusin, Liliana Szyszka-Sommerfeld, Małgorzata Idzior-Haufa, Małgorzata Pobudek-Radzikowska, Krzysztof Woźniak, Agata Czajka-Jakubowska

**Affiliations:** ^1^Department of Orthodontics and Temporomandibular Disorders, Poznan University of Medical Sciences, Poznan, Poland; ^2^Department of Maxillofacial Orthopedics and Orthodontics, Pomeranian Medical University in Szczecin, Szczecin, Poland; ^3^Laboratory for Propaedeutics of Orthodontics and Facial Congenital Defects, Pomeranian Medical University in Szczecin, Szczecin, Poland

**Keywords:** oral behaviors, orofacial pain, orthodontic patients, TMD, parafunctions

## Abstract

**Introduction:**

Few studies have evaluated oral behaviors in patients undergoing orthodontic treatment. While occlusal and non-occlusal parafunctions may significantly contribute to TMD symptoms, their frequency in orthodontic patients remains unclear. This study aimed to assess the occurrence of oral parafunctions, TMD pain, and headaches in this population.

**Materials and methods:**

The study included patients undergoing orthodontic treatment with fixed appliances. The Fonseca Anamnestic Index, DC/TMD Axis I, and Oral Behavior Checklist (OBC) questionnaires were used to assess the occurrence of TMD pain and oral parafunctions.

**Results:**

The study included 152 patients. 59.2% of the study participants were women, the mean age was 20.01 (SD 6.89). The painful form of TMD was found in 23.7% of the study participants, with headaches in 26.4% (with TMD-attributed headaches in 13.2%). The mean score on the OBC questionnaire was 18.96 (SD 8.89) and 25% of patients had high-risk grade of oral behaviors.

**Conclusion:**

Patients experiencing myalgia, arthralgia, and headaches had notably higher OBC scores. Patients undergoing orthodontic treatment should be screened for oral parafunctions and TMD pain.

## 1 Introduction

Oral parafunctional behaviors involve mouth actions beyond its primary functions of chewing, swallowing, and speaking (1). The relationship between these behaviors and temporomandibular disorders (TMD) has been frequently studied, with research sometimes revealing a positive link between parafunctions and TMD. However, other studies have found no such association (1–6).

One of the most used questionnaires for assessing oral parafunctions is the Oral Behavior Checklist (OBC), a component of Axis II Diagnostic Criteria for Temporomandibular Disorders (DC/TMD). The OBC is a 21-item self-report questionnaire designed to evaluate the frequency and nature of parafunctional oral behaviors, including teeth grinding and clenching, nail biting, tongue thrusting, gum chewing, yawning, singing, etc. It asks individuals to indicate how often they engage in each specific behavior (7, 8).

From an orthodontic perspective, it is essential to note that certain parafunctional habits, such as nail biting and abnormal tongue function, can cause fixed retention failure (9). What’s interesting is that parafunction, like chewing gum, can have dual effects: on the one hand, they may cause hypertrophy of the masseter muscle, while on the other, orthodontists often recommend them as an effective alternative to analgesics for alleviating orthodontic pain (10, 11). According to Keela et al. (3), intensifying various parafunctional habits increases the muscle’s sustained tonic contraction or heightens the temporomandibular joint (TMJ) load, prolonging pain symptoms and contributing to chronicity. Cioffi et al. (12) claim that higher OBC values were statistically significantly positively associated with experimental pain induced by placing orthodontic separators.

While orthodontic treatment does not cause TMD symptoms, myalgia, arthralgia, or other orofacial pain symptoms may still arise during treatment (13, 14). Due to the duration of orthodontic treatment, patients may experience TMD symptoms during or after treatment and blame orthodontists, leading to potential legal issues (14). Michelotti et al. (14) emphasize that it is expected to encounter patients with a history of orofacial pain in everyday orthodontic clinical practice. Consequently, conducting a routine TMD-related examination before starting orthodontic treatment seems essential.

In the available literature, great interest is placed on assessing pain of orthodontic origin (15). Still, there are few studies evaluating the occurrence of occlusal and non-occlusal parafunctions among patients undergoing orthodontic treatment (16). Therefore, although oral behaviors might significantly contribute to developing TMD and pain, the frequency of these behaviors in orthodontic patients has yet to be fully assessed.

The study aimed to assess the occurrence of oral parafunctions and TMD pain among patients undergoing orthodontic treatment.

## 2 Materials and methods

The study involved patients receiving orthodontic treatment with fixed appliances. The inclusion criteria were active orthodontic treatment and informed consent to participate in the study. Patients with a history of head or craniofacial injuries, chronic neurological or rheumatological diseases, or those taking neuropsychiatric medication were excluded from the study. Also, patients using intermaxillary elastics were not included in the study, as their use could influence occlusal forces and muscle activity. This research utilized the Polish version of the Fonseca questionnaire and evaluated the presence of TMD-related pain based on DC/TMD Axis I criteria. Additionally, the study examined the occurrence of oral parafunctions using the polish version of Oral Behavior Checklist, which is part of Axis II of the DC/TMD (17–19).

The Fonseca Anamnestic Index (FAI) questionnaire consists of 10 questions. The alternatives to these questions are: “yes,” “sometimes,” and “no” with values of 10 points, 5 points, and 0 points, respectively. The scores of the 10 questions are added up and the results are interpreted. The overall test score ranges from 0 to 100. The FAI assesses the presence or absence of symptoms caused by TMD and their severity (mild, moderate, and severe) (19–21).

Axis I DC/TMD questionnaire allows for the following TMD diagnoses (17). The main subtypes of pain-related temporomandibular disorders are myalgia, arthralgia and headaches attributed to TMD. In contrast, TMJ disc displacement, degenerative joint disease, and subluxation are the primary subtypes of intra-articular TMD conditions. Our research investigated the painful manifestations of temporomandibular disorders (TMD).

Myalgia refers to muscle-originating pain that is influenced by jaw movement, function, or parafunction, and can be reproduced during provocation tests of the masticatory muscles (17). Arthralgia is joint-originating pain similarly impacted by jaw movement, function, or parafunction, with replication of the pain during provocation testing of the temporomandibular joint (TMJ). Headache attributed to TMD refers to a headache localized to the temple region, caused by pain-related TMD, and is influenced by jaw movement, function, or parafunction, with the headache reproduced during provocation testing of the masticatory system (17).

The Oral Behaviors Checklist is a self-report scale to identify and quantify the frequency of jaw overuse behaviors (8). The OBC is a self-reported 21-item questionnaire that quantifies the frequency of oral behaviors OB performed during the preceding month. In the questionnaire are two groups of activities: during sleep and during waking hours. According to the DC/TMD scoring manual of self-report instruments, oral behaviors OBC total score can be used divided into 3 levels: no behavior (score 0), low risk behavior (score 1–24), and high risk behavior (score 25–84) (3).

### 2.1 Statistic

Statistical analyses were performed using SPSS v23 software. Descriptive statistics were applied to calculate the mean values, standard deviations (SD), and the minimum and maximum values of the demographic variables. The Kolmogorov–Smirnov test was used to assess the normality of the data distribution. To compare differences between independent groups, the t-test and Mann–Whitney U test were utilized. A *p*-value of less than 0.05 was considered statistically significant in all tests.

The study received the approval of the Bioethics Committee of the Medical University of Poznań KB-370-24.

## 3 Results

The study included 152 patients undergoing orthodontic treatment with fixed appliances. Of these, 59.2% were women, and the average age was 20.01 years (SD 6.89).

The mean score on the OBC questionnaire was 18.96 (SD 8.89). None of the subjects were found to have no behavior, 61.3% were found to have low risk, and 20.4% were found to have high risk of OB. The mean score on the Fonseca Anamnestic Index was 18.22 (SD 14.18), with 52.6% showing no TMD, 36.8% exhibiting mild TMD, and 10.5% presenting with moderate TMD. None of the patients had severe TMD.

According to the DC/TMD questionnaire, 23.7% of the patients had painful TMD, with 21.1% diagnosed with myalgia and 5.3% with arthralgia. Headaches contributed to TMD were observed in 13.5% of the patients. Table 1 presents the categorization of patients by gender, diagnosis based on the Fonseca and DC/TMD Axis I questionnaires, and the mean OBC score for each group.

**Table 1:** Associations between mean OBC score and gender and TMD diagnosis.

Variable	Category	*n*	Mean OBC	*p*-value
Gender	Male	62	16.65 (SD 7.02)	*p* = 0.005
	Female	90	20.56 (SD 9.69)	
TMD diagnoses according to FAI	No TMD	80	14.95 (SD 6.66)	*p* < 0.001
	Mild TMD	56	21.00 (SD 8.22)	
	Moderate TMD	16	31.88 (SD 5.97)	
TMD Diagnoses according to DC/TMD	TMD-Free	108	16.54 (SD 6.95)	*p* < 0.001^*^
	Painful TMD	36	26.83 (SD 10.36)	
	Non-painful TMD	8	16.25 (SD 3.24)	
Headaches	No headache	112	16.46 (SD 7.29)	*p* < 0.05
	Headache contributed to TMD	20	29.30 (SD 11.09)	
	Other headache	20	22.60 (SD 5.60)	

*Tukey’s HSD indicated that the mean OBC score of the pain-related TMD group was statistically significantly higher than the mean OBC scores of other subgroups (*p* ≤ 0.05), and there was no difference between TMD-free and non-painful TMD group.

Women, as well as patients with moderate TMD based on the FAI, painful TMD according to the DC/TMD criteria, and those experiencing headaches contributed to TMD, exhibited statistically higher scores on the OBC questionnaire.

The subsequent phase of our research was to determine which oral behaviors were most common among orthodontic patients. The findings, presented in Table 2, show that the most frequent behaviors were sleeping in a position that puts pressure on the jaw (Q2) – 81.6%, chewing gum (Q13) – 80.3%, leaning with hand on the jaw (Q15) – 78.9%, eating between meals (Q17) – 77.6%, and yawning (Q20) – 63.8%.

**Table 2:** Summary of OBC-21 survey responses provided as the number and percentage of participants.

	0	1	2	3	4	Positive score
	None of the time	<1 Night / Month	1–3 Nights / Month	1–3 Nights / Week	4–7 Nights / Week	
Q1	128 (84.2)	0	12 (7.9)	6 (3.9)	6 (3.9)	24 (15.8)
Q2	28 (18.4)	2 (1.3)	6 (3.9)	28 (18.4)	88 (57.9)	**124 (81.6)**
	None of the time	A little of the time	Some of the time	Most of the time	All of the time	
Q3	128 (84.2)	12 (7.9)	12 (7,9)	0	0	24 (15.8)
Q4	86 (56.6)	20 (13.2)	36 (23.7)	8 (5.3)	2 (1.3)	66 (43.4)
Q5	98 (64.5)	20 (13, 2)	18 (11.8)	10 (6.6)	6 (3.9)	54 (35.5)
Q6	106 (69.7)	26 (17.1)	16 (10.5)	0	4 (2.6)	46 (30.3)
Q7	132 (86.8)	10 (6.6)	2 (1.3)	4 (2.6)	4 (2.6)	20 (13.2)
Q8	120 (78.9)	10 (6.6)	10 (6.6)	4 (2.6)	8 (5.3)	32 (21.1)
Q9	120 (78.9)	14 (9.2)	8 (5.3)	4 (2.6)	6 (3.9)	32 (21.1)
Q10	96 (63.2)	18 (11.8)	20 (13.2)	10 (6.6)	8 (5.3)	56 (36.8)
Q11	98 (64.5)	20 (13.2)	26 (17.1)	4 (2.6)	4 (2.6)	54 (35.5)
Q12	80 (52.6)	24 (15.8)	32 (21.1)	10 (6.6)	6 (3.9)	72 (47.4)
Q13	30 (19.7)	40 (26.3)	54 (35.5)	18 (11.8)	10 (6.6)	**122 (80.3)**
Q14	148 (97.4)	0	4 (2.6)	0	0	4 (2.6)
Q15	32 (21.1)	26 (17.1)	60 (39.5)	22 (14.5)	12 (7.9)	**120 (78.9)**
Q16	70 (46.1)	32 (21.1)	10 (6.6)	26 (17.1)	14 (9.2)	82 (53.9)
Q17	34 (22.4)	32 (21.1)	60 (39.5)	18 (11.8)	8 (5.3)	**118 (77.6)**
Q18	82 (53.9)	26 (17.1)	22 (14.5)	20 (13.2)	21 (1.3)	70 (46.1)
Q19	69 (45.4)	27 (17.8)	38 (25)	11 (7.2)	7 (4.6)	83 (55.6)
Q20	55 (36.2)	30 (19.7)	44 (28.9)	18 (11.8)	5 (3.3)	**97 (63.8)**
Q21	106 (69.7)	28 (18.4)	12 (7.9)	6 (3.9)	0	46 (30.3)

Bold values represent the five most frequently occurring oral behaviors.

The final step of the study involved examining which oral behaviors were associated with painful forms of TMD in orthodontic patients. Those with myalgia exhibited a higher frequency of behaviors such as teeth clenching and grinding during sleep (Q1), teeth grinding and clenching during wakefulness and holding teeth together (Q3, Q4, Q5), tensing jaw muscles and holding the jaw to the side (Q6, Q7), pressing the tongue against the teeth (Q8), and sustained talking (Q21). Among patients with arthralgia, the behaviors included teeth grinding and clenching while awake (Q3, Q4). For patients with headaches attributed to TMD, the associated behaviors were teeth clenching and holding the teeth together during wakefulness (Q4, Q5), tensing jaw muscles (Q6), biting the tongue, cheeks, or lips (Q10), maintaining a rigid jaw position and resting the jaw on the hand (Q11, Q15), and yawning (Q20). The above results are presented in Table 3.

**Table 3:** Mean scores and standard deviations of responses to individual OBC questions categorized by painful-TMD diagnoses based on DC/TMD criteria.

	Myalgia			Artralgia			Headache		
	Present	Absent		Present	Absent		TMD headache	No headache	
Q1 Clench or grind teeth (sleep)	1.31 (1.59)	0.20 (0.71)	***p* < 0.001**	1.00 (1.85)	0.40 (0.99)	*p* = 0.395	1.00 (1.65)	0.30 (0.84)	*p* = 0.080
Q2 Sleep position pressure jaw	3.38 (1.33)	2.85 (1.56)	*p* = 0.63	2.75 (1.75)	2.97 (1.52)	*p* = 0.735	3.50 (1.24)	2.82 (1.54)	*p* = 0.650
Q3 Grind teeth (waking)	0.75 (0.84)	0.10 (0.39)	***p* < 0.001**	1.00 (0.76)	0.19 (0.54)	***p* = 0.019**	0.40 (0.68)	0.20 (0.55)	*p* = 0.218
Q4 Clench teeth (waking)	1.56 (1.08)	0.62 (0.95)	***p* < 0.001**	2.00 (1.31)	0.75 (1.00)	***p* = 0.031**	1.80 (1.44)	0.66 (0.90)	***p* = 0.002**
Q5 Press, touch or hold teeth together	1.31 (1.42)	0.57 (1.01)	***p* = 0.008**	1.75 (1.91)	0.67 (1.07)	*p* = 0.154	2.20 (1.64)	**0.55 (0.89)**	***p* < 0.001**
Q6 Hold, tighten or tense muscles without clenching	1.25 (1.36)	0.28 (0.55)	***p* < 0,001**	1,50 (1,77)	0,43 (0,78)	*p* = 0,133	1,50 (1,47)	**0,27 (0,55)**	***p* < 0,001**
Q7 Hold or jut jaw forward/to the side	0.87 (1.43)	0.12 (0.49)	***p* = 0.006**	1.25 (1.75)	0.22 (0.73)	*p* = 0.142	0.50 (1.24)	0.20 (0.64)	*p* = 0.295
Q8 Press tongue against teeth	1.31 (1.59)	0.27 (0.77)	***p* = 0.001**	1.00 (1.31)	0.46 (1.07)	*p* = 0.286	1.00 (1.45)	0.34 (0.90)	*p* = 0.062
Q9 Place tongue between teeth	0.75 (1.22)	0.35 (0.91)	*p* = 0.091	1.25 (1.75)	0.39 (0.92)	*p* = 0.209	0.80 (1.44)	0.28 (0.73)	*p* = 0.133
Q10 Bite…tongue. cheeks or lips	1.12 (1.29)	0.70 (1.18)	*p* = 0.098	0.50 (0.92)	0.80 (1.22)	*p* = 0.398	1.90 (1.74)	0.64 (1.02)	***p* = 0.005**
Q11 Hold jaw in rigid or tense position	0.75 (1.11)	0.63 (1.00)	*p* = 0.592	0.75 (0.88)	0.65 (1.03)	*p* = 0.772	1.10 (1.07)	**0.43 (0.76)**	***p* = 0.013**
Q12 Hold between the teeth or bite objects	1.06 (1.27)	0.90 (1.14)	*p* = 0.514	1.25 (1.75)	0.92 (1.13)	*p* = 0.611	1.40 (1.54)	0.89 (1.12)	*p* = 0.172
Q13 Use chewing gum	1.18 (0.89)	1.70 (1.16)	***p* = 0.009**	1.50 (1.20)	1.59 (1.13)	*p* = 0.828	1.60 (1.14)	1.54 (1.11)	*p* = 0.818
Q14 Play musical instrument involves mouth/jaw	0	0.07 (0.36)	*p* = 0.298	0	0.06 (0.32)	*p* = 0.636	0	0.07 (0.37)	*p* = 0.395
Q15 Lean with your hand on the jaw	2.00 (1.19)	1.63 (1.17)	*p* = 0.127	2.5 (1.20)	1.67 (1.17)	*p* = 0.092	2.40 (1.39)	**1.48 (1.07)**	***p* = 0.010**
Q16 Chew food on one side only	1.50 (1.57)	1.15 (1.36)	*p* = 0,255	1,75 (1,91)	1,19 (1,39)	*p* = 0,442	1,70 (1,45)	1,07 (1,33)	*p* = 0,083
Q17 Eating between meals	1.75 (0.98)	1.52 (1.15)	*p* = 0,256	2,00 (1,31)	1,54 (1,11)	*p* = 0,362	1,80 (0,89)	1,37 (1,13)	*p* = 0,070
Q18 Sustained talking	1.81 (1.26)	0.67 (0.99)	***p* < 0.001**	0.75 (0.89)	0.92 (1.17)	*p* = 0.625	0.80 (1.01)	0.91 (1.19)	*p* = 0.663
Q19 Singing	0.69 (0.99)	0.57 (1.06)	*p* = 0.551	1.00 (1.31)	0.57 (1.03)	*p* = 0.389	1.30 (1.65)	0.52 (0.91)	*p* = 0.052
Q20 Yawning	1.75 (1.16)	1.58 (1.04)	*p* = 0.466	2.00 (1.51)	1.60 (1.04)	*p* = 0.480	2.20 (1.20)	**1.42 (0.97)**	***p* = 0.012**
Q21 Hold telephone between your head/shoulders	0.44 (0.88)	0.47 (0.79)	*p* = 0.865	0.75 (1.39)	0.44 (0.76)	*p* = 0.556	0.40 (0.94)	0.48 (0.81)	*p* = 0.717

Bold values are statistically significant difference.

## 4 Discussion

This study aimed to examine the relationships between self-reported oral parafunctional behaviors and TMD-related pain in orthodontic patients with fixed appliances, as numerous studies have hypothesized that certain oral behaviors may be linked to a higher risk of developing TMD (4, 5, 22, 23).

Statistically significant associations were observed between these behaviors, gender, TMD diagnosis, and different types of pain-related TMD. In our patient population, the prevalence of painful TMD was 23.7%, which was slightly lower than the rates reported in Barbosa et al.’s (5) study among students (27.9%) and Lovgren et al.’s (6) study (27.8%).

In our studies, patients without TMD had an OBC score of 14.95 according to the FAI diagnosis and 16.54 points according to the DC/TMD diagnosis. These scores fall between the results reported by Reda et al. (4), who found an OBC score of 13.3 in TMD-free individuals, and Barbosa et al. (5), who reported a mean total OBC score of 17.7 for TMD-free participants. Despite the differences, our findings and these studies indicate that the mean total OBC scores for TMD-free individuals correspond to a low-risk grade on the OBC scale. Reda pointed out that a low-risk OBC grade is commonly observed in TMD-free populations, whereas higher OBC scores are more frequently associated with TMD patients (4).

Our study found that women had significantly higher OBC scores than men, contrasting with findings from other research by van der Meulen et al. (1), Michelotti et al. (2), and Khawaja et al. (24). However, Karaman and Buyuk (25) observed a similar pattern as ours in his study involving patients seeking orthodontic treatment, and Barbosa et al. (5) also noted higher OBC scores among women. This suggests that there may be a gender-related predisposition that warrants further investigation, highlighting the importance of designing future studies to better understand why women may be more susceptible to higher OBC scores.

It is worth noting that no statistically significant differences in OBC scores were observed between patients without TMD and those with non-painful TMD, aligning with the findings of Donnarumma and Khawaja (24, 26). However, our patients with painful TMD demonstrated a higher risk of engaging in oral behaviors. Interestingly, while some researchers did not identify this association (3, 26, 27), Keela et al. (3) reported a correlation between OBC scores and pain chronicity. Differences in study group selection may explain these conflicting results.

Our study included patients undergoing orthodontic treatment. Crossbite and longitudinal changes in occlusion may be associated with TMD symptoms, while the number of symptoms does not seem to be affected by orthodontic treatment (28). Karaman and Buyuk (25) did not find any correlation between malocclusion (Class I, II, or III) and OBC scores. Antoun et al. (16) examined differences in the frequency of oral behaviors between hyperdivergent and normodivergent patients at various stages: before, during, and after treatment. The results showed no significant differences in self-reported oral behaviors between normodivergent and hyperdivergent individuals, suggesting no association between vertical facial form and habitual muscular activity. However, their research highlighted that the stage of treatment might have influenced the pattern of oral behaviors (16).

In our orthodontic patients, the most common oral behavior was sleeping in a position that exerts pressure on the jaw (81.6%), and another was eating between meals (77.6%). However, neither of these behaviors was associated with painful forms of TMD, highlighting that not all oral parafunctional activities significantly contribute to pain intensity (1). Their high frequency might explain the lack of association with the most prevalent behaviors—since nearly everyone engages in these behaviors, they may lack the variability needed to reveal meaningful differences or associations (5).

The next most frequent activity was chewing gum. In our research we found an interesting link between chewing gum and myalgia, with patients who chewed gum less frequently reporting masticatory muscle pain more often. More than 80% of participants reported chewing gum, which, as noted by Guo et al. (11), is a non-invasive, cost-effective, and convenient way to alleviate orthodontic pain without affecting bracket stability. Based on this, chewing gum can be recommended as an alternative to analgesics for managing orthodontic discomfort. Although chewing gum is often considered a parafunctional activity that may lead to masseter hypertrophy, studies using near-infrared spectroscopy have offered further insights into its effects on muscle hemodynamics (29, 30). For example, women in the TMD group showed greater severity of TMD symptoms, lower orofacial myofunctional scores, and reduced oxygen extraction during unilateral chewing of a silicon device compared to healthy controls. Ferreira et al. (29) also found that oxygen extraction rates were significantly associated with the severity of TMD and orofacial myofunctional disorders. Additionally, Tsutsui et al. (30) observed that 1 month of gum chewing training led to improvements in masseter oxygen dynamics during clenching and recovery, enhancing muscle aerobic capacity and showing a downward trend in pain scores. These findings align with our study results, emphasizing the complex relationship between chewing habits, muscle function, and pain management.

Among the five most common oral behaviors (OB), leaning with a hand on the jaw and yawning were associated with TMD headaches. While the literature lacks descriptions of a link between leaning on the jaw and TMD headaches, the association with yawning is well-documented. Yawning is frequently observed in clinical contexts as a symptom linked to migraine attacks, with repetitive yawning reported in 45.4% of migraine patients, particularly during the premonitory phase and headache episodes (31). Furthermore, yawning can sometimes trigger severe headaches in individuals, a condition known as “primary yawning headache,” which has been noted in patients with cranial neuralgia and related disorders (32).

Our study found a significant prevalence of oral behaviors among orthodontic patients, highlighting the importance of early identification. Detecting these parafunctional habits promptly enables clinicians to educate patients and work toward reducing their frequency. Early identification of high-risk patients may allow for preventive interventions, reducing the risk of pain-related complications during orthodontic treatment. As noted by Xu et al. (33), combining patient education with physical therapy can effectively alter oral behaviors in individuals with TMD.

Due to the high prevalence of orofacial pain, every dental clinician, not just orthodontists, should be aware of how to recognize it and understand the available treatment options (34).

Limitations. One limitation of this study is the lack of differentiation between other reported headaches, such as migraines or tension-type headaches, as no formal neurological assessment was conducted. Another limitation is the cross-sectional design, which did not account for the duration of orthodontic treatment - a factor that may influence the development of TMD symptoms and oral parafunctions. Additionally, the study did not categorize patients based on malocclusion type, which could be relevant in evaluating the relationship between occlusion, parafunctional behaviors, and TMD symptoms.

## 5 Conclusion

Our findings indicate a high prevalence of oral behaviors in the studied population. Parafunctional oral behaviors have been recognized as a potential contributor to the signs and symptoms of painful temporomandibular disorders (TMD). Screening patients undergoing orthodontic treatment could facilitate early interventions, reducing the risk of pain developing during treatment.

## Data Availability

The raw data supporting the conclusions of this article will be made available by the authors, without undue reservation.
